# Effect of social marketing on the knowledge, attitude, and uptake of pap smear among women residing in an urban slum in Lagos, Nigeria

**DOI:** 10.1186/s12905-022-01620-5

**Published:** 2022-02-14

**Authors:** Tope Olubodun, Mobolanle Rasheedat Balogun, Kofoworola Abimbola Odeyemi, Akin Osibogun, Oluwakemi Ololade Odukoya, Adekunbiola Aina Banjo, Sandra Esse Sonusi, Ayodeji Bamidele Olubodun, Oluwatoyin Olanrewaju Progress Ogundele, Duro Clement Dolapo

**Affiliations:** 1 Department of Community Health and Primary Care, Lagos University Teaching Hospital, Lagos, Nigeria; 2 Department of Community Health and Primary Care, College of Medicine, University of Lagos, Lagos, Nigeria; 3 Department of Anatomic and Molecular Pathology, College of Medicine, University of Lagos, Lagos, Nigeria; 4 Department of Anatomic and Molecular Pathology, Lagos University Teaching Hospital Lagos, Lagos, Nigeria; 5 Olabisi Onabanjo University Teaching Hospital, Sagamu, Ogun State Nigeria; 6 Ladiya Hospital, Zaria, Kaduna State Nigeria; 7 Leverage Consulting Limited, Abuja, Nigeria

**Keywords:** Social marketing, Cervical cancer, Cervical cancer screening, Pap smear, Knowledge, Attitude, Uptake, Health education, Behaviour change

## Abstract

**Introduction:**

Nigeria has a low uptake of cervical cancer screening and is one of the five countries that represent over half of the global burden of deaths from cervical cancer. Social marketing principles can be used to design and implement interventions to increase uptake of cervical cancer screening. This study assessed the effect of a social marketing intervention on the knowledge, attitude, and uptake of pap smear among women residing in an urban slum in Lagos State, Nigeria.

**Materials and methods:**

This was a quasi-experimental study. The intervention arm consisted of 140 women recruited from Ago-Egun Bariga community and the control arm consisted of 175 women recruited from Oto-Ilogbo extension community. Social marketing intervention was instituted in the intervention group. Data analysis was done using IBM SPSS Statistics version 20 and Stata version 16.0. Between groups comparisons and within groups comparisons were done using bivariate analysis with Chisquare, Students *t* test and Paired *t* test as appropriate.

**Results:**

In both the intervention and control groups, the mean knowledge score of cervical cancer was low at baseline (0.0 ± 0.3 and 0.1 ± 0.9 respectively). In the intervention group, there was a significant increase in mean knowledge score to 15.1 ± 3.7, post-intervention (*p* < 0.001). In both groups, the mean attitude score of cervical cancer was low at baseline (27.1 ± 0.8 in the intervention group and 27.2 ± 1.4 in the control group). In the intervention group, there was a significant increase in mean attitude score to 36.5 ± 4.8, post-intervention (*p* < 0.001). In both the intervention and control groups, uptake of pap smear was low at baseline (0.0% and 0.6%, respectively). In the intervention group, there was a significant increase in uptake of pap smear to 84.3%, post-intervention (*p* < 0.001). There was no statistically significant change in knowledge, attitude or uptake of pap smear in the control group, post-intervention.

**Conclusion:**

This study demonstrated that social marketing intervention can be successful in improving knowledge, attitude, and also the uptake of pap smear, even in settings where these are abysmally low. It is recommended that social marketing intervention be employed as a strategy for improving cervical cancer screening among women residing in slums.

## Introduction

Globally, cervical cancer is ranked the second most common female cancer in women of reproductive age and the fourth most common cancer in women [1]. In 2018, there was an estimated 570,000 new cases and 311,000 deaths worldwide [2, 3]. About 85% of the global burden occurs in the less developed regions and these regions account for nine out ten (87%) cervical cancer deaths worldwide. [4, 5] The age-standardized rate for cervical cancer in Nigeria is 36.0 per 100,000 [5]. Nigeria, India, China, Brazil, and Bangladesh represent over 50% of the global burden of cervical cancer deaths [6]. In Nigeria, cervical cancer is the second leading cause of female cancer deaths, after cancer of the breast [7]. Many cases of cervical cancer present late in Nigeria and nationwide screening programmes are lacking [7].

In Nigeria, the majority of studies done on cervical cancer screening have shown very low uptake of screening. Studies conducted among populations with good knowledge of cervical cancer, such as among nurses, also showed poor uptake of screening [8, 9]. Several health education intervention studies carried out in Nigeria to improve cervical cancer screening have failed to achieve their aim. These studies were only able to improve knowledge of cervical cancer screening, but this did not translate to improved practice [10–12].

Essential schemes are required to improve the practice of screening as knowledge alone is insufficient to promote acceptance and use of cervical cytological screening tests across many settings in Nigeria [11]. There is a need for public health interventions that not only produce an increase in knowledge of cervical cancer prevention but also the practice of screening [13]. Strategies to address cervical cancer prevention, need to provide access to services and address socio-cultural, economic and other barriers to cervical cancer screening [13]. Such interventions can be designed and implemented by employing the principles of social marketing.

Social marketing is an approach used to develop activities that are aimed at changing or maintaining people’s behaviour for the benefit of individuals and society as a whole [14]. Social marketing applies marketing principles and techniques to create, communicate, and deliver value in order to influence target audience behaviours that benefit society as well as the target audience [15]. Social marketing technique has been used extensively in international health programmes, especially for contraceptives and oral rehydration therapy (ORT) [16]. In developing countries, the use of social marketing has been expanded to HIV prevention, malaria control and treatment, point-of-use water treatment, on-site sanitation methods and the provision of basic health services [16].

The planning process of social marketing takes the consumer focus into account by addressing the elements of the "marketing mix." This refers to decisions about the conception of a Product, Price, Place and Promotion. These are often called the "Four Ps" of social marketing [17].

The "product" is what is being marketed, such as a certain behaviour one is trying to change. In this research, the behaviour to change is the practice of cervical cancer screening (pap smear) [18]. The "Price" refers to what the consumer must do in order to obtain the social marketing product [17]. This cost may be monetary, or it may instead require the consumer to give up intangibles, such as time or effort, or to risk embarrassment and disapproval such as husband’s disapproval [17]. If the costs outweigh the benefits for an individual, the perceived value of the offering will be low and it will be unlikely to be adopted [17].

"Place" is largely where and when the target audience will be encouraged to perform the desired behaviour and/or to obtain tangible products or services associated with the campaign [15]. As in commercial marketing, place can be regarded as the delivery system or a distribution channel for a social marketing campaign [15]. The "Place" has to be as convenient and pleasant as possible for the customer to engage in the targeted behaviour and access related products and services [15]. "Promotion" is the last of the "4 Ps," and the one most easily associated with social marketing. Promotion is the advertising done [18]. Promotion consists of the integrated use of advertising, public relations, promotions, media advocacy, personal selling and entertainment vehicles. Some methods include media events, editorials, short message service (SMS), workshops, talks and coupons [17].

The majority of people who reside in slums are of low socioeconomic status, which is shown to be associated with an increased risk of cervical cancer as a result of poor access to reproductive health services as well as a higher prevalence of risky sexual behaviours [19]. People of the low socioeconomic class usually have low awareness of cervical cancer, low perception of risk, and poor practice of cervical cancer screening [20, 21].

This study employed social marketing techniques to provide a set of interventions aimed at creating an enabling environment in addition to providing information. This is intended to increase knowledge and positive attitudes towards cervical cancer screening, as well as increase pap smear uptake in the intervention community. Findings from this study will guide policymakers and programme designers in their decisions and choices regarding the promotion of cervical cancer screening.

## Methods

### Study area

This study was conducted in Lagos State, South-West Nigeria. Though the smallest in area of Nigeria's 36 states, Lagos State is arguably the most economically important state of the country [22]. Lagos has a population estimated at 21 million in 2016, which makes it the most populous state in Nigeria and the largest city in Africa [23] Pap smear services are only available in a few public healthcare facilities in Lagos. Some private hospitals and diagnostic centres also offer pap smear services to clients.

There has been a rapid development of slums in Lagos as a result of rapid urbanization and rural–urban migration [24]. Lagos has 192 identified slum communities [25]. Urban slums are settlements, neighbourhoods, or city regions that cannot provide the basic living conditions necessary for its inhabitants, to live in a safe and healthy environment [26]. The United Nations Human Settlements Programme (UN-HABITAT) defines a slum settlement as one that cannot provide any of the following basic living characteristics: Durable housing of a permanent nature that protects against extreme climate conditions; Sufficient living space, which means no more than three people sharing the same room; Easy access to safe water in sufficient amounts at an affordable price; Access to adequate sanitation in the form of a private or public toilet shared by a reasonable number of people; Security of tenure that prevents forced evictions [26].

### Intervention community

Ago-Egun Bariga is a slum settlement located in Bariga Local Council Development Agency in the city of Lagos. The inhabitants are mainly of the Egun tribe with a few people of the Yoruba tribe. Ago-Egun Bariga is overcrowded and is lacking in basic social amenities. There is no pipe-borne water or drainage system. Human faeces are deposited into the water near the residence. The majority of the houses are built with wooden planks on the water surfaces. Others are old buildings made of mud-plastered walls and dilapidated zinc roofing.

### Control community

Otto-Ilogbo extension is under the administration of the Lagos Mainland Local Government area. It is located around a large refuse dump located in between the Otto community and Ilogbo community, hence its name, Otto-Ilogbo extension. Its inhabitants are mainly Yoruba and Igbo petty traders and artisans who live freely amongst each other. There is no form of town planning in Otto-Ilogbo extension and the houses are numerous. Most of the houses are made of wooden planks and dilapidated zinc. There is no drainage system or pipe-borne water in the community and community members mostly practice open defecation. Otto-Ilogbo extension is grossly overcrowded.

The distance between the study community and the control community is about 20 km by road. Both communities are located within different local governments, which are not contiguous in location. The commercial activities of both slum communities are also not directly related. This minimizes the chances of interaction between control and intervention arms, as well as spill-over effects of the intervention.

### Study design

This study is a quasi-experimental controlled study with pre and post design, aimed at determining the effect of social marketing intervention on the knowledge, attitude and uptake of pap smear among women residing in an urban slum.

### Study population

The study population consisted of women aged 21–65 years who reside in the two selected urban slums in Lagos—Ago-Egun Bariga (Intervention community) and Oto-Ilogbo extension (Control community). The inclusion criteria for the study were women who had resided in the slum for at least 1 year and women that had been married/cohabiting/sexually active. The exclusion criteria were women who were too sick to attend the health education sessions/cervical cancer screening, women who were pregnant during the course of the study.

### Sample size determination

The study was based on the hypothesis that at post-intervention, the intervention group would have at least 20% (0.20) improvement in the knowledge, attitude, and uptake of Pap smear screening for cervical cancer [10]. The sample size was determined using the formula for the comparison of proportions [27].

$${\text{n }} = \frac{{\left[ {{\text{Z}}_{\alpha } + {\text{Z}}_{\beta } } \right]^{{2}} \left\{ {\left[ {{\text{P}}_{{1}} \left( {{1} - {\text{P}}_{{1}} } \right)} \right] \, + \, \left[ {{\text{P}}_{{2}} \left( {{1} - {\text{P}}_{{2}} } \right)} \right]} \right\}}}{{\left[ {{\text{P}}_{{1}} {-}{\text{ P}}_{{2}} } \right]^{{2}} }}$$

Prevalence (P_2_) of women who had undergone cervical cancer screening in a previous study was 13.3% [28].

The expected prevalence (P_1_) after the intervention was 33.3%. The calculated minimum sample size was 66 for each group. Because multistage sampling was employed, design effect was taken into consideration and the minimum sample size was multiplied by 2. The sample size then came to 132. After compensating for attrition, with an attrition rate of 30%, the sample size calculated was 188 for each group.

### Sampling method

A multistage sampling method was used in selecting study participants. In the first stage, two slums from the list of slums in Lagos State were selected using a computer-generated table of random numbers. The sampling frame included all the 192 identified slum communities in Lagos. The first slum selected-Ago-Egun Bariga- was the intervention community while the second selected—Otto-Ilogbo extension—was the control community.

In the second stage, each community was divided into five clusters based on the arrangement of houses and forty houses were selected from each cluster. The "spin the bottle" EPI-derived technique (a technique derived by EPI research Inc for cluster sampling in household surveys) was used in selecting the houses in each cluster [29]. The index house in each cluster was selected by spinning a bottle in the middle of the cluster. The bottle was observed to see where its tip points; the house whose front door was closest to the tip was the index house. The next house chosen was the one whose front door was closest to the index one.

In the third stage, where there was more than one eligible female in a house, the respondent was selected by simple random sampling by balloting. In the event that there was no eligible female in a selected house, it was excluded and the next house was selected.
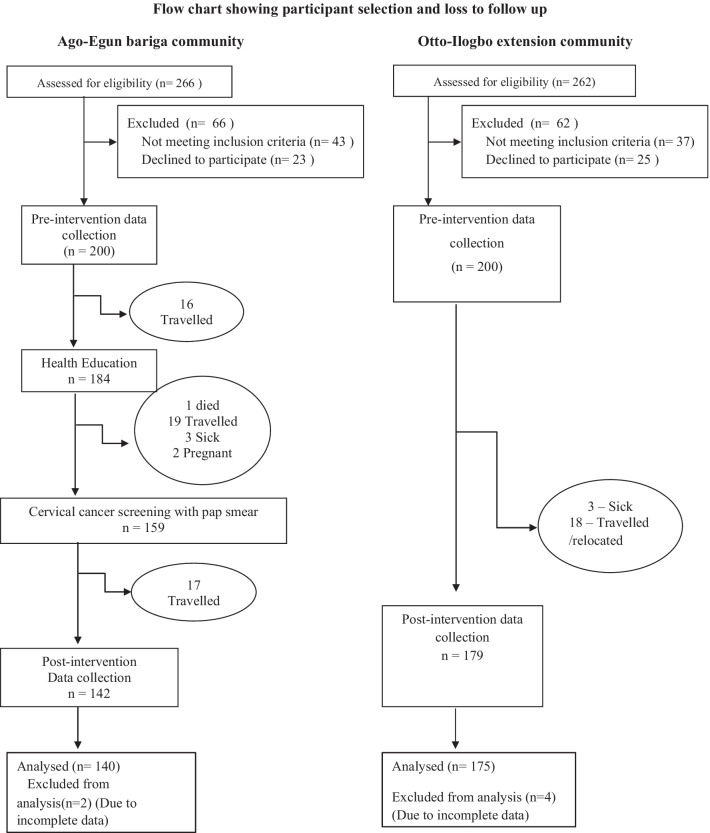


### Pre-intervention phase

#### Baseline data collection

Data was collected using interviewer-administered questionnaires. The questionnaire was adapted from previous studies. [12, 30–32] It contained questions on the socio-demographic characteristics of respondents, knowledge of cervical cancer (which include; ever heard of cervical cancer, symptoms and risk factors of cervical cancer, prevention of cervical cancer—screening and HPV vaccination), knowledge of pap smear (which include; ever heard of pap smear, how frequently the test should be done, women eligible for testing), attitude towards cervical cancer (which include Likert statements on perceived susceptibility to cervical cancer, perceived severity of cervical cancer and perception of screening), and uptake of pap smear. The questionnaires were pretested in another slum not used for the study and adjustments were made. Four trained female research assistants with post-secondary degrees collected data.

#### Focused group discussions

Focused group discussions (FGD) were conducted among women age 21–65 in Ago Egun Bariga—the intervention community prior to the commencement of the intervention. Homogenous groups of 8–10 women of similar age were used. The focused group discussion was aimed at gaining a deeper understanding of the women’s perceptions about cervical cancer screening, barriers and recommendations, in order to aid the design and implementation of the social marketing intervention.

Findings from the qualitative survey guided the social marketing intervention. The respondents wanted the test to be free or largely subsidized and wanted female providers. Many said they will require the consent of their husbands to undergo the test. Also, the majority of the discussants wanted the test to be carried out in their community. The women suggested the use of a megaphone, SMS and health education sessions to promote the intervention.

### Social marketing intervention

#### Application of the principles of social marketing

Eight ‘benchmarks’ describe the key concepts and principles of social marketing and include Customer orientation, Behavioural focus, Exchange, Developing insight, Competition analysis, Theory, Segmentation, and Methods Mix [33]. *Customer orientation* involves ‘seeing things through the customer’s eyes’. It involves understanding where the customer is starting from, their knowledge, attitudes and beliefs, and their social context [34]. Prior to the intervention, these were assessed using interviewer-administered questionnaires and focus group discussions. *Behavioural focus* implies that the intervention is focused on influencing specific behaviours, not just knowledge, attitudes, and beliefs [35]. In this regard, our study aimed to increase the uptake of pap smear, and not just increase knowledge and improve attitudes.

*Exchange* considers benefits and costs of adopting and maintaining a new behaviour; maximizes the benefits and minimizes the costs to create an attractive offer [35]. In this study, we provided pap smear services and explained the benefits of pap smear to the participants. We also reduced the costs by providing the pap smears free and making it easily accessible within the community. Spouses of the participants were also educated to reduce husband’s disapproval (an intangible cost). *Developing insight* involves developing a deeper understanding of what is or is not likely to engage a target audience or motivate them in relation to a particular behaviour [33]. In our study, focus group discussions conducted before the intervention helped to understand potential enabling factors and barriers to change.

*Competition analysis* in social marketing leads to the identification of countervailing forces and the systematic development of strategies to reduce the impact of these external and internal competitive forces [33]. Some of the external countervailing forces we identified in our study were spouse’s disapproval, cost of the test, and distance to the testing site. These were addressed in the intervention. Internal countervailing forces included poor knowledge and attitudes towards cervical cancer screening and we addressed these by providing health education.

*Theory* involves using behavioural theories to understand target behaviour and ‘inform’ the intervention [36]. *The health belief model* guided our intervention [37]. During the health education sessions, women received adequate information on the risk factors of cervical cancer so they can understand that all sexually active women are at risk of the disease and thus develop healthy perceptions of personal susceptibility (*Perceived Susceptibility*) [37]. The health education also provided information on the seriousness and consequences of cervical cancer in terms of symptoms and complications, ill-health and suffering, time lost from work, and economic difficulties (*Perceived Severity*) [37]. Our intervention also addressed the benefits of pap smear in detecting precancerous changes early, before cancerous changes manifest (*Perceived Benefits*).

We assessed barriers to cervical cancer screening using questionnaires and focus group discussions and addressed them. The perceived barriers identified included religious and cultural barriers, spouse’s disapproval, feeling embarrassed, and cost. The support of religious leaders, husbands, and traditional leaders was sought. The religious and traditional leaders helped promote the pap smear services by speaking at our health education sessions. The barrier of cost was addressed by making the pap smears free. Feelings of embarrassment were minimized by using only female service providers (*Perceived Barriers*). The cues provided in this study included SMS to remind the women about the date and time of the pap smears, and banners displayed strategically in the community in English, Yoruba, and Egun languages (*Cues to Action*). Undergoing pap smear does not require much effort from the client and we provided information on how to prepare for a pap smear test and what to expect in the health education sessions (*Self-efficacy*).

*Segmentation* involves assigning people to groups that exhibit similar characteristics, beliefs values, and behaviours in order to, develop specifically targeted interventions, designed to help them change behaviour [33]. In this study, we tried to identify segments of the population with similar characteristics. Using the socio-demographic characteristics, most of the women were married, of the Egun tribe, were of the Christian religion, had no formal education, and had low incomes. Hence they could not be segmented along these lines. Also, almost all the women had poor knowledge, poor attitude and all had not had cervical cancer screening in the past. The participants also had similar motivational factors e.g. cost, distance, and spousal support. The women were more similar in characteristics than different.

#### Marketing mix

**Fig. 1:**
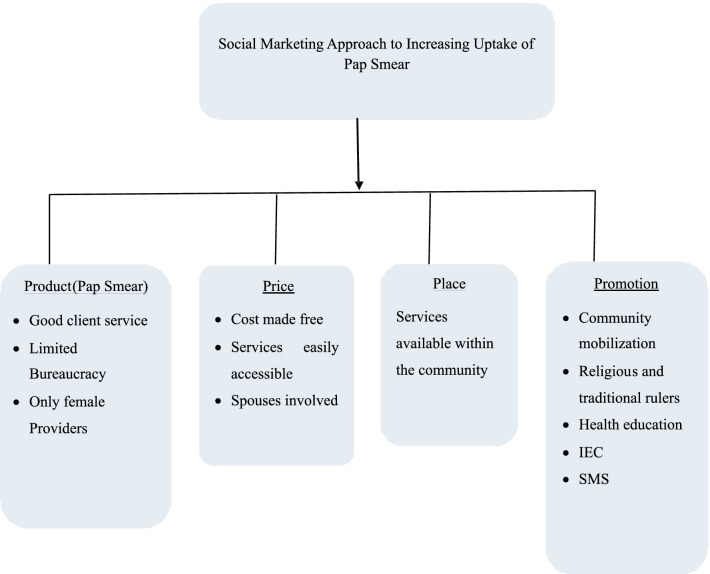
Social marketing framework

*Product* The providers were trained and supervised to be warm and receptive. They were trained to be courteous and to re-assure the women about the procedure and also to minimize discomfort during the procedure. Only female providers were recruited to provide pap smears as this was a recommendation from the focus group discussion. Bureaucracy, which is often the case in government hospitals where pap smear is provided was limited (Fig. 1).

*Price* Pap smear services were offered free of charge. The clients did not need to pay for transportation, as the location of the services was within the community. This study had sensitization meetings for husbands to enable the spouses to understand the importance of pap smears and hence reduce disapproval (Fig. 1).

*Place* Services were made available within the community (Fig. 1). The place used was suitable and comfortable for the women and the procedure. Adequate privacy was ensured. Also, responses from the FGD had suggested a location within the community. The venue used was the clinic of a traditional birth attendant in the community, who is certified and registered with the State government ministry of health. The centre was re-painted to make it more appealing for the intervention.

*Promotion* Six health education sessions were organized for the women with each woman attending one health education session. A meeting was also carried out to educate their husbands on cervical cancer and pap smear. The health belief model guided the content of the health education sessions and information education communication (IEC) materials—handbills and banners. Contents of the health education included: a description of cervical cancer, its burden, risk factors, symptoms, complications, and prevention. The health education also included details on the Pap smear test, its importance, how frequently it should be done, and who should have it done. A brief description was also given of what to expect during the test (Fig. 1).

As part of the promotion, community mobilization was carried out and three community mobilizes were sought amongst key people in the community who understood the local dialect. Religious clerics and community leaders also publicly showed support for the intervention through speeches. Information, Education, and communication (IEC) materials in form of handbills and banners were used and reminder SMS’s were sent out periodically. The positioning statement for the social marketing intervention was *‘Cervical Cancer Is Real, Get a Pap Smear Test Today’*. This was written in English, Egun, and Yoruba Languages and was boldly displayed on all banners, handbills, and t-shirts.

### Post-intervention phase

After the intervention, the same respondents who were interviewed at the beginning of the study were interviewed again using the same questionnaire to assess their knowledge, attitude, and uptake of Pap smear. The interviews were conducted for both the intervention group and the control group. Women in the control group had health education sessions on cervical cancer and free pap smears after the study, for ethical reasons.

### Timelines

The study duration was 7 months from pre-intervention data collection to post-intervention data collection. The Health education sessions lasted for 6 weeks and afterwards, pap smears were made available in the community for a period of 3 months. There was a period of 4 months between the health education sessions, and the post-intervention data collection.

### Data management and analysis

Data entry and cleaning were done using Microsoft Excel 2010. Data was then imported and analysed using IBM SPSS Version 20.0 (Armonk, NY: IBM Corp). Stata Version 16 (College Station, TX: Stata Corp LLC) was used for difference in difference analysis. Categorical data were summarized using frequencies and proportions. Numerical data were summarized using means and standard deviations, median and interquartile range. Observations with incomplete data were excluded from analysis.

A scoring method was developed to quantify the respondents’ knowledge of cervical cancer and pap smear and also their attitude towards cervical cancer and pap smear. For the knowledge scoring, ten knowledge questions were scored. Knowledge questions scored include; ever heard of cervical cancer, symptoms of cervical cancer known, risk factors of cervical cancer known, if cervical cancer can be prevented, cervical cancer prevention measures known, ever heard of tests that detect cervical cancer early, does detecting cervical cancer early improve treatment outcome, type of cervical cancer screening tests known, ever heard of pap smear, and how often pap smear tests should be done. Correct responses were awarded a point each and incorrect responses no point. Some questions allowed multiple correct responses. The maximum attainable score was 32 while the lowest attainable score was 0. The mean knowledge score was calculated.

There were nine questions assessing attitudes of women towards cervical cancer and pap smear on a Likert scale. The questions were; Cervical cancer is a severe disease, I could be susceptible to cervical cancer, I cannot have cervical cancer because I don’t have multiple sexual partners, I cannot have cervical cancer because I believe I am spiritually protected, Cervical cancer is a death sentence, Chances of curing cervical cancer are better when the disease is discovered at an early stage, Cervical cancer can be prevented from occurring, Cervical cancer screening is important, and I am comfortable with having a cervical cancer screening test. The highest score for each question was 5 while the lowest score was 1. The possible range of scores was 5–45. The mean attitude score was calculated.

Intergroup comparisons (intervention and control group comparisons) were made at baseline. Within-group comparison of the intervention group and the control group, before and after intervention was also done. Comparison of proportions between two groups was done using Pearson’s chi-squared test or Fisher’s exact test as appropriate. For numerical data, independent sample T-test was used to compare across the two groups while paired T-test was used to compare each group before and after. Repeated measures analysis was also used to assess within and between group changes.

Difference-in-difference (DID) analysis was used to estimate intervention effects, adjusting for biases that could be the result from permanent differences between the groups (pre-existing differences), as well as biases from comparisons over time in the intervention group that could be the result of trends due to other causes of the outcome (time trends) [38]. The difference-in-difference is an early quasi-experimental strategy for estimating causal effects [39]. Difference-in-difference is a useful technique to use when randomization on the individual level is not possible [38]. The DID estimate is defined as the difference in the average outcome in the intervention group before and after the intervention minus the difference in the average outcome in the control group before and after the intervention [39]. DID analysis was done using linear random-effects regression with an interaction term between study arm and study period. [39]

The level of significance was set at 5%. Associations or differences were considered statistically significant if *p* values were less than or equal to 0.05.

### Ethical considerations

Approval for this study was obtained from the health research and ethics committee of the Lagos University Teaching Hospital with approval number: ADM/DCST/HREC/APP/2028. Written informed consent was obtained. All methods were carried out in accordance with the relevant guidelines and regulations. e.g., the Declaration of Helsinki.

## Results

The study was completed with 140 women in the intervention group and 175 women in the control group out of 188 women in each of the groups at baseline. This represented a response rate of 74% in the intervention group and 93.1% in the control group.

The mean age for the intervention group was 34.2 ± 10.4 years, and 35.0 ± 10.1 years for the control group. Both groups were similar in respect to their ages and there was no statistically significant difference between their ages (*p* = 0.217). The majority of the respondents in the intervention group had no formal education (85.7%), while the majority in the control group had secondary education as their highest level of education (65.1%). The observed difference in proportions was statistically significant (*p* < 0.001). The median monthly income was $16 ($10–$16) in the intervention group and $30 ($20–$30) in the control group. The difference observed was statistically significant (*p* < 0.001) (Table 1).

**Table 1:** Sociodemographic characteristics of respondents

Sociodemographic characteristics	Frequency (%)			Statistic	*p* value
	Intervention	Control	Total		
	n = 140	n = 175	n = 315		
Age (in years)					
21–30	63 (45.0%)	76 (43.4%)	139 (44.1%)	5.714^F^	0.217^F^
31–40	50 (35.7%)	50 (28.6%)	100 (31.7)		
41–50	18 (12.9)	39 (22.3)	57 (18.1)		
51–60	5 (3.6)	7 (4.0)	12 (3.8)		
60–65	4 (2.9)	3 (1.7)	7 (2.2)		
Mean ± SD	34.2 ± 10.4	35.0 ± 10.1		0.625^T^	0.533^T^
Marital status					
Single	1 (0.7)	11 (6.3)	12 (3.8)	29.265^F^	**< 0.001**^**F**^
Married/cohabiting	133 (95.0)	134 (76.6)	267 (84.8)		
Divorced/separated	0 (0.0%)	19 (10.9)	19 (6.0)		
Widowed	6 (4.3)	11 (6.3)	17 (5.4)		
Ethnicity					
Yoruba	7 (5.0)	109 (62.3)	116 (36.8)	348.672^F^	**< 0.001**^**F**^
Hausa	0 (0.0)	4 (2.3)	4 (1.3)		
Igbo	0 (0.0)	47 (26.9)	47 (14.9)		
Egun	132 (94.3)	1 (0.6)	133 (42.2)		
Others	1 (0.7)	14 (8.0)	15 (4.8)		
Religion					
Christianity	137 (97.9)	128 (73.1)	265 (84.1)	35.576^X^	**< 0.001**^**F**^
Islam	3 (2.1)	47 (26.9)	50 (15.9)		
Level of education					
No formal education	120 (85.7)	21 (12.0)	141 (44.8)	194.298^F^	**< 0.001**^**F**^
Primary education	11 (7.9)	39 (22.3)	50 (15.9)		
Secondary education	9 (6.4)	114 (65.1)	123 (39.0)		
Tertiary education	0 (0.0)	1 (0.6)	1 (0.3)		
Postgraduate education	0 (0.0)	0 (0.0)	0 (0.0)		
Occupation					
Unemployed	10 (7.1)	19 (10.9)	29 (9.2)	7.835^F^	**0.037**^**F**^
Unskilled	115 (82.1)	121 (69.1)	236 (74.9)		
Semi-skilled	15 (10.7)	32 (18.3)	47 (14.9)		
Skilled	0 (0.0)	3 (1.7)	3 (1.0)		
Average monthly income					
≤ Ṩ36	109 (77.9)	113 (64.6)	222 (70.5)	6.598^X^	0.013^X^
> Ṩ36	31 (22.1)	62 (35.4)	93 (29.5)		
Median (IQR)	$16 ($10–$16)	$30 ($20–$30)		8423.5^U^	**< 0.001**^U^

Statistically significant in bold

^U^Mann Whitney—U

^T^Independent sample T-test

^F^Fishers exact

^X^Chi-square

There was an increase in mean knowledge score in the intervention group from 0.0 ± 0.3 to 15.1 ± 3.7 and the difference was statistically significant (*p* < 0.001). In the control group, there was no significant increase in knowledge (*p* = 0.096). DID estimate, for overall knowledge score was statistically significant and positive which indicates that in the intervention group, there was an increase in overall knowledge score compared with the control group after adjusting for potential differences in time trends and at baseline (Table 2).

**Table 2:** Paired T-test and repeated measures analysis showing changes in respondents’ mean knowledge score

	Intervention group	Control group
Pre-intervention	0.0 ± 0.3	0.1 ± 0.9
Post-intervention	15.1 ± 3.7	0.2 ± 1.4
T (*p* value)	− 48.80 (*p* < **0.001)**	− 1.68 (*p* = 0.096)
D	15.1	0.1
DID estimate (95% CI, *p* value)	15.0 (95% CI 14.3–15.6, *p* < **0.001)**	
*F*^*KS*^ (*p* value, partial eta squared)	2945.452 (*p* < **0.001,** η^2^ 0.094)	
*F *^KS*Arm^ (*p* value, partial eta squared)	2900.951 (*p* < **0.001,** η^2^ 0.903)	
*F*^*Arm*^ (*p* value, partial eta squared)	1862.257 (*p* < **0.001,** η^2^ 0.856)	

*T* Paired T-test, *d* difference of means, *DID* difference-in-difference

***F***^***KS***^ repeated measures ANOVA value (of knowledge score over time)

***F***^***Arm***^ repeated measures ANOVA value (of knowledge score between study arm)

***F ***^KS*Arm^ repeated measures ANOVA value (of interaction term between knowledge score and study arm)

**η**^**2**^ Partial eta squared Statistically significant in bold

The results of repeated measures ANOVA indicated there was a significant increase in knowledge scores over time Wilks’ Lambda = 0.096 *F*^*KS*^ = 2945.452, *p* < 0.001, η^2^ 0.094. The change in knowledge score over time, showed a difference between study arms (as indicated by a significant interaction effect between knowledge score and study arm) Wilks’ Lambda = 0.097 *F*^KS*Arm^ = 2900.951, *p* < 0.001, η^2^ 0.093. There was a significant difference in knowledge scores across groups *F*^*Arm*^ = 1862.257, *p* < 0.001, η^2^ 0.856 (Table 2).

There was an increase in mean attitude score in the intervention arm from 27.2 ± 1.4 to 36.5 ± 4.8 and the difference observed was statistically significant. In the control arm, there was no statistically significant increase in mean attitude score, before and after the intervention (*p* = 0.068). DID estimate for mean attitude score was statistically significant and positive, which indicates that in the intervention arm, overall attitude score significantly increased among the intervention arm compared with the control arm after adjusting for potential differences in time trends and at baseline (Table 3).

**Table 3:** Paired T-test and repeated measures analysis showing changes in respondents’ mean attitude score

	Intervention group	Control group
Pre-intervention	27.2 ± 1.4	27.2 ± 1.4
Post-intervention	36.5 ± 4.8	27.3 ± 1.6
T (*p* value)	− 22.96 (*p* < **0.001)**	− 1.84 (*p* = 0.068)
d	9.3	1.1
DID estimate (95% CI, *p* value)	9.3 (95% CI 8.5–10.1, *p*|< **0.001)**	
*F*^*KS*^ (*p* value, partial eta squared)	661.542 (*p* < **0.001,** η^2^ 0.679)	
*F *^KS*Arm^ (*p* value, partial eta squared)	645.614 (*p* < **0.001,** η^2^ 0.673)	
*F*^*Arm*^ (*p* value, partial eta squared)	403.679 (*p* < **0.001,** η^2^ 0.563)	

The results of repeated measures ANOVA indicated there was a significant increase in attitude scores over time Wilks’ Lambda = 0.0321 *F*^*KS*^ = 661.542, *p* < 0.001, η^2^ 0.679. The change in attitude score over time, showed a difference between study arms (as indicated by a significant interaction effect between attitude score and study arm) Wilks’ Lambda = 0.0327 *F*^KS*Arm^ = 645.614, *p* < 0.001, η^2^ 0.673. There was a significant difference in attitude scores across arms *F*^*Arm*^ = 403.679, *p* < 0.001, η^2^ 0.563 (Table 3).

In the intervention group, the uptake of cervical cancer screening increased from 0.0 to 84.3%. This difference was statistically significant. In the control arm, there was no statistically significant increase in uptake of cervical cancer screening. DID estimate for uptake of pap smear was statistically significant and positive, which indicates that uptake of pap smear improved significantly among the intervention arm compared with the control arm after adjusting for potential differences in time trends and at baseline (Table 4).

**Table 4:** Uptake of pap smear among respondents, before and after intervention

	Intervention group	Control group
Pre-intervention	0 (0.0)	1 (0.6)
Post-intervention	118 (84.3)	1 (0.6)
X^2^ (*p* value)	203.95 (*p* < **0.001)**	0.01 (*p* = 1.000)
D	84.3	0.0
DID estimate (95% CI, *p* value)	84.3 (95% CI 0.8–0.9, *p* < **0.001)**	

## Discussion

In this study, the mean knowledge score significantly increased in the intervention group, while in the control group, however, the mean knowledge score remained similar. This was consistent with the findings of a study conducted among teachers in Birnin Kebbi, northwest Nigeria, after a health education intervention, in which the overall mean knowledge score increased significantly in the intervention group [10]. Many health education intervention studies [10–12, 40, 41] have also reported increased knowledge of cervical cancer, after the intervention. Similarly, this study also has a health education component and did find an increase in knowledge of cervical cancer.

The finding of an increase in the mean attitude score of the intervention group was similar to that of a health education intervention study among rural women in Ogun State, Southern Nigeria, in which there was an increase in the mean attitude score in the intervention group [42]. The finding was also consistent with that of another health education intervention study by Adamu et al., in which the mean attitude score increased from 35.4 ± 10.3 to 52.8 ± 3.4 and the difference was statistically significant [10].

In this social marketing intervention study, there was a marked rise in uptake of pap smear in the intervention group, and the observed difference was statistically significant. In the control group, however, uptake of pap smear did not change. In this study, the social marketing intervention was successful at increasing the uptake of pap smear. On the contrary, several health education interventions carried out in Nigeria [10–12], only succeeded in increasing knowledge and attitude towards cervical cancer, but there was no statistically significant increase in uptake. In a health education intervention study among teachers in Birnin Kebbi, North West, Nigeria, there was an increase in the mean knowledge score of cervical cancer from 25.5% ± 10.5 to 57.2% ± 20.7 in the intervention group (*p* < 0.001). In the intervention group, mean attitude score also rose from 17.1% ± 6.3 to 28.0% ± 12.8 (*p* < 0.001) [10]. However, uptake of pap smear was low in the intervention group at baseline and post-intervention with no statistically significant change [10].

In the Birnin Kebbi teachers study, respondents were given a free coupon for pap test to be carried out at the federal teaching hospital in the state [10]. Though the test was free, uptake was still low, and many respondents complained of dislike for the test because the majority of the sample collectors were males [10]. There was also a problem of long waiting times at the hospital which was discouraging and also, lack of ease of access to the federal teaching hospital [10]. These discouraging factors were addressed in the present study as only female sample collectors were recruited, and to improve geographical access, the test was made available within the community. These could have contributed to the success reported in our study. Shyness to use the services domiciled in the community was not a problem because the location chosen was private and it was a place where women in the community usually deliver their babies.

In a health education intervention study carried out among market women in Lagos, though there in the intervention group, there was a significant rise in awareness of pap test from 6.9 to 56.6%, uptake of the test did not change significantly (1.1% pre-intervention to 1.7% post-intervention) [11]. In another health education intervention study carried out among market women in Niger State, there was a significant increase in awareness of pap smear from 1.1 to 34.1% while uptake of pap smear only rose from 1.1 to 3.4%, an increase that was not statistically significant [12].

In the above studies among market women in Lagos and market women in Niger State, health education sessions were held and IEC materials were distributed [11, 12]. However, the test was not subsidized or free and the respondents were to visit hospitals for the test. These did not increase uptake of the test and poor financial and geographical access may have been responsible for the low uptake. In the present study, however, the pap smear test was offered free and was made available within the community.

In a health education intervention study conducted by Abiodun et al. among women in rural Odogbolu and Ikenne LGAs in Ogun State, southwest Nigeria, there was a statistically significant increase in knowledge, attitude, as well as uptake (even though small) of cervical cancer screening [42]. In the Ogun study, VIA services were available in three health centres in Odogbolu LGA and four VIA centers were available in Ikenne LGA. There was a 4.0% increase in the uptake of cervical screening among the intervention group, post-intervention [42]. In the present study, however, there was an 84.3% increase in uptake in the intervention group, post-intervention. This could indicate that social marketing intervention (interventions that address the 4 ‘Ps’—Product, Price, Place, and Promotion), when compared to health education alone may be able to cause higher levels of change in the uptake of cervical cancer screening. Our intervention also had an intensive community mobilization component as part of the ‘promotion’. This could have also contributed to the large uptake post-intervention.

In a health education intervention study among Samoan women recruited from Samoan churches in the U.S., after the intervention, 61.7% of women in the intervention group compared to 38.3% in the control group self-reported having obtained a Pap smear and the difference was statistically significant [43]. In a health education intervention study that took place among Hispanic women aged 18–65 years in Mexico, uptake of pap smear increased significantly in the intervention group. Uptake of pap smear was 65.0% in the intervention group and 36.0% in the control group, 6 months post-intervention, and the difference was statistically significant [44].

The observation that health education intervention seems to have better results at improving pap smear uptake in the US study [43] and Mexico study [44], when compared to the Nigerian studies [10–12] may suggest that different women across different countries may respond differently to health education interventions, based on factors which may range from cultural factors, religious beliefs, financial barriers and geographical barriers, etc. Also, in the US study, since Samoan women from churches were selected, positive reinforcement and group-based dynamics could have contributed to the increased uptake of pap smear. It may be that either the women leaders in the churches may have reinforced the education programme, hence resulting in behaviour change.

Similar to the Nigerian health education studies, in a health education intervention study carried out in rural Kenya, after the follow-up period, the mean knowledge score increased significantly in the intervention group compared to the control group. There was, however, no difference in the change in acceptance of screening between the two arms [45].

The importance of cervical cancer screening in reducing the incidence and burden of cervical cancer cannot be over-emphasized. The vast difference in cervical cancer incidence in developing versus developed countries is a reflection of the poor screening uptake in the majority of developing countries [13]. This study was carried out with the aim of increasing cervical cancer screening, in addition to increasing knowledge and attitude towards screening. This was achieved using a well-designed social marketing intervention.

### Strength and limitations of the study

This study adds appreciably to the body of knowledge as this is one of the very few interventional studies focusing on the use of social marketing to increase uptake of pap smear. A limitation of the study based on the prospective study design was loss to follow up. The sample size was thus increased by an attrition factor. Also, the contact details of the respondents were collected at the beginning of the study, in a confidential manner to allow for tracing of respondents during post-intervention data collection. The questionnaires were not validated. Another limitation of the study was a paucity of studies in African counties or similar settings to compare findings.

## Conclusion

This study demonstrated that a social marketing intervention was successful at improving knowledge, attitude, and uptake of pap smear even in settings where these were abysmally low. It is recommended that social marketing intervention be employed on larger scales to improve screening for cervical cancer among Nigerian women. Efforts toward improving cervical cancer screening uptake among women should aim at addressing the financial, geographical, and socio-cultural barriers to screening and not just merely providing information.

Areas of further research needed for future studies will include cost evaluation of social marketing interventions aimed at improving uptake of cervical cancer screening. In addition, as social marketing in this study involved multiple interventions, future studies may be carried out to include a subset of these interventions and evaluate if these will still produce effective results at lower costs.

## Data Availability

The datasets used and/or analysed during the current study are available from the corresponding author on reasonable request.

## Abbreviations

DID: Difference-in-difference
FGD: Focused group discussions
HIV: Human immunodeficiency virus
IEC: Information Education Communication materials
ORT: Oral rehydration therapy
SMS: Short message service
SPSS: Statistical Product and Service Solutions
US: United States
